# Fibroblast growth factor 21 and growth differentiation factor 15 as biomarkers for oxidative stress in children with methylmalonic acidemia

**DOI:** 10.1186/s12887-026-06719-4

**Published:** 2026-03-27

**Authors:** Maha A. Abd Elbaky, Mohamed Shehata, Khalil Abdelkhalek, Marian Y. Girgis, Mohamed A. Elmonem

**Affiliations:** 1 Department of Clinical and Chemical Pathology, Faculty of Medicine, Cairo University, Cairo, Egypt; 2 Department of Pediatrics, Faculty of Medicine, Cairo University, Cairo, Egypt

**Keywords:** FGF21, GDF15, Glutathione, Oxidative stress, Organic acidemia

## Abstract

**Background:**

Methylmalonic acidemia (MMA) is a rare autosomal recessive inborn error of metabolism commonly presenting in infancy with episodes of metabolic acidosis that can lead to considerable morbidity and early mortality. Oxidative stress is a major aspect in the pathogenesis of MMA. We aimed to evaluate the clinical utility of fibroblast growth factor 21 (FGF21) and growth differentiation factor 15 (GDF15) and correlate their levels with established biomarkers of oxidative stress, such as reduced glutathione (GSH) and reduced/oxidized (GSH/GSSG) glutathione ratio in MMA children in a clinical setting.

**Methods:**

Nineteen MMA pediatric patients (median 5.7; IOR 2.9–7.3 years) were recruited. Thirty age and sex matched healthy children were further recruited as a comparator group. Total glutathione, reduced glutathione, FGF21 and GDF15 were assayed for both cohorts.

**Results:**

Total and reduced glutathione were significantly reduced in MMA patients compared to controls (*P* < 0.001 for both), while FGF21 and GDF15 were significantly elevated (*P* = 0.037 and < 0.001, respectively). Both FGF21 and GDF15 had a significant negative correlation with GSH/GSSG ratio (*r*=–0.256; *P* = 0.038 and *r*=–0.367; *P* = 0.005, respectively); however, only GDF15 had a negative correlation with both total and reduced glutathione (*r*=–0.443; *P* < 0.001 and *r*=–0.459; *P* < 0.001, respectively). GDF15 had a better performance in the ROC curve analysis; AUC = 0.805 vs. 0.668 for FGF21, while FGF21 was a significant predictor of eGFR in the linear regression analysis.

**Conclusions:**

FGF21 and GDF15 were both significantly increased in the studied MMA pediatric patients and had strong correlations with established oxidative stress biomarkers. Since both are more stable during storage and easier to assay than glutathione based indicators, their use as candidate biomarkers for oxidative stress in MMA children can be considered.

## Introduction

Methylmalonic acidemia (MMA) is an autosomal recessive inborn error of metabolism (IEM) disorder. It often causes multisystem injury but mainly affects the central nervous system [1]. MMA is one of the most common organic acidemias, with an incidence ranging from 1:48,000 to 1:61,000 in North America, and 1:26,000 in China [2]. Although its incidence is not established in Egypt, MMA was one of the most commonly encountered IEM disorders diagnosable by tandem mass spectrometry in Egyptian children among the clinically suspected [3, 4] and in a limited pilot newborn screening study conducted in 25,000 healthy newborns [5].

Several genes have been identified to cause MMA; the most common is *MMUT* gene, which constitutes approximately 60% of patients. Other causative genes include *MMAA*,* MMAB*,* MMACHC* and *MCEE* [6].

The clinical manifestations of MMA are variable and lack specificity compared to other organic acidemias. In the neonatal period, patients can present with acute and commonly severe metabolic symptoms, such as metabolic acidosis, feeding difficulties, vomiting and poor general condition, and may develop neurological symptoms as well as non-neurological manifestations, such as hematological abnormalities, hydrocephalus, and pulmonary hypertension [2, 7]. When metabolic decompensation occurs later during infancy, neurological features usually dominate with lack of visual focus, hypotonia, irritability, lethargy and coma. With disease progression, untreated children usually exhibit failure to thrive, neurological deterioration, commonly associated with cardiac and kidney complications [2].

The toxic accumulation of organic acid metabolites in various cells often leads to oxidative stress. These metabolites directly promote the generation of free radicals, which deplete the cell’s natural antioxidant defenses. The accumulation of these toxic metabolites impairs mitochondrial function and generates excessive reactive oxygen species that contribute to neurological and systemic damage [8].

Fibroblast growth factor 21 (FGF21) is a member of the fibroblast growth factor (FGF) gene family that is mainly expressed in the liver, brain and pancreas [9]. Serum level of FGF21 is affected by altered lipid metabolism, and is commonly increased in patients with mitochondrial oxidative stress and can be used as a predictor of disease progression [10, 11]. Although FGF21 is now considered as an established biomarker in mitochondrial respiratory chain disorders it is also considered as a very promising biomarker for the mitochondrial dysfunction in organic acidemias, particularly methylmalonic and propionic acidemias [12, 13].

Growth differentiation factor 15 (GDF15) is a cell stress-responsive cytokine and a divergent member of the transforming growth factor-β (TGF-β) superfamily. It is mainly expressed in the bladder, kidney, colon, stomach, liver, gall bladder, pancreas, lung and endometrium [14, 15]. Its expression increases under pathological or stress conditions, such as diabetes, smoking, surgery, exercise, cancer, metabolic associated fatty liver disease, cardiovascular and kidney diseases [16].

In this prospective cross-sectional study, we aimed to investigate the roles of FGF21 and GDF15 and compare their values as potential biomarkers for the screening of oxidative stress and clinical severity in children with methylmalonic acidemia.

## Subjects and methods

### Patients

Nineteen MMA children were recruited from the neurometabolic and nutrition clinics at Cairo University Children’s Hospital during the period from January 2022 to October 2024. Thirty age and sex matched healthy control children were also recruited for the comparison of various oxidative stress biomarkers.

Recruited pediatric patients all had a biochemical confirmation of MMA in the form of elevated propionyl carnitine (C3) in expanded metabolic screening by tandem mass spectrometry followed by elevation of the characteristic organic acids in urine by gas chromatography mass spectrometry. Patients less than three months or more than 18 years and patients with chronic liver disease were excluded from the study.

The study was conducted in accordance with the declaration of Helsinki for studies involving human participants and was approved by the institutional research ethics committee at Faculty of Medicine, Cairo University (Approval code:#MD-299-2021). Written informed consents to participate and to publish were obtained from parents/legal guardians of all participants.

### Methods

Detailed history, clinical, laboratory and radiological data of all recruited children were obtained from the clinics’ data records with special emphasis on age, sex, antenatal and perinatal history, symptoms and signs of the patient, the age of onset of symptoms, similar cases in the family and parental consanguinity. A detailed neurological examination was conducted including cognition, motor, sensory, cerebellar, gait and reflexes. Systemic examination also included, cardiac, chest and abdominal examination. Laboratory investigations performed were complete blood count, blood gases, kidney and liver function tests and special investigations for IEM such as blood ammonia, lactate, amino acids and acylcarnitines analysis in blood spots by tandem mass spectrometry [17] and urinary organic acid profile by gas chromatography mass spectrometry [18]. Radiological investigations were variable according to the main presentations of every patient and were determined by the clinician as appropriate. They included cranial/abdominal ultrasonography (US), brain magnetic resonance imaging (MRI) and echocardiography.

All participants in this study were sampled for the analysis of total and reduced glutathione, FGF21 and GDF15 in serum. All analyses were carried out at the Chemical Pathology Department, Faculty of Medicine, Cairo University.

Three milliliters of blood were collected in sterile vaccutainer tubes without preservatives and were centrifuged to collect serum. Sera of patients were stored frozen at − 80◦C until analysis. Total glutathione (T-GSH) was performed by the CheKine™ total glutathione colorimetric assay kit (Abbkine, code #KTB1670; Wuhan, Hubei, China). Human reduced glutathione (GSH) was conducted by the ELISA kit of bioassay technology laboratory (code #EA0142Hu; Yangpu, Shanghai, China). Total fibroblast growth factor-21 and growth differentiation factor-15 were assayed by using ELISA kits of ABclonal (codes #RK00084 and #RK00086, respectively; Wuhan, Hubei, China). Results were calculated by creating standard curves for each analyte as per the manufacturers’ protocols.

### Statistical analysis

Data entry, processing and statistical analyses were carried out using MedCalc v.20 (MedCalc, Ostend, Belgium). Data were summarized as means and standard deviations (mean ± SD) for parametric numerical data, while median and inter-quartile ranges (IQR) were used for non-parametric numerical data. Frequencies were used for non-numerical data. Mann-Whitney’s U-test was used to assess the difference of quantitative variables between two study groups, while Chi-Square test was used for categorical variables. Association of the tested oxidative biomarkers with the continuous dependent variables “estimated GFR” and “hemoglobin” was performed through multivariate linear regression analysis after adjusting for age, sex, duration of illness, ammonia, lactate, fasting glucose and ALT levels. The oxidative biomarkers were also associated with the dichotomous variable “presence of complications” (renal, cardiac and hepatic) through multivariate logistic regression analysis adjusted for the same confounders. ROC (receiver operating characteristic) curves were used to evaluate the diagnostic performance measures. The Youden-Index was used to find the optimal cutoff point in the ROC curve analysis. *P*-values < 0.05 were considered statistically significant.

## Results

### Demographic and clinical data

Forty-nine pediatric subjects were recruited in this study including 19 children from 19 unrelated families with the verified diagnosis of methylmalonic acidemia confirmed by initial elevation of propionyl carnitine in blood spots and methylmalonic acid among others in urinary organic acids profile. MMA patients were 7 males (36.8%), and 12 females (63.2%) with median age of 5.7 years (IOR 2.9–7.3 years). Control group included 30 apparently healthy children with median age 5.5 years (IQR 4-9.8 years). They were 14 males (46.7%) and 16 females (53.3%) with no significant difference regarding age and sex against MMA patients.

The average age of onset of manifestation in MMA children was 5.3 ± 5 months and the average age at diagnosis was 10.9 ± 10.3 months. 47.4% of patients had similar cases in the family, (73.7%) of families were consanguineous, previous sibling death was recorded in (42.1%) of families, and (31.6%) of mothers had a history of unexplained abortions (Table 1).

**Table 1:** Demographic, clinical and laboratory data of the MMA children and healthy controls

Variable	Patients group (*n* = 19)	Control group (*n* = 30)	*P* value
Demographic characteristics			
Age (years)	5.7 (2.9–7.3)	5.5 (4-9.8)	0.361
Sex (male/female)	7/12	14/16	0.498
Age of onset (months)	5.6 (1.25-6)		
Age at diagnosis (months)	14.4 (2.5–16)		
Similar cases	9 (47.4%)		
Consanguinity	14 (73.7%)		
Sibling death	8 (42.1%)		
History of abortion	6 (31.6%)		
Clinical Characteristics			
Neurological manifestations	12(63.1%)		
GIT manifestations	12 (63.1%)		
Metabolic manifestations	9 (47.4%)		
Failure to thrive	3 (15.7%)		
Delayed motor development	9 (47.4%)		
Intellectual disability	6 (31.6%)		
Hypotonia	3 (15.7%)		
Extrapyramidal manifestations	2 (10.5%)		
Chronic kidney disease	3 (15.7%)		
Cardiomyopathy	2 (10.5%)		
Optic atrophy	1 (5.3%)		
Hepatomegaly	1 (5.3%)		
Laboratory findings			
Hb (g/dL)	10.2 (9.9–11)	12.1 (11.1–13)	< 0.001
PLT (10^3^/µL)	307 (252–389)	345 (289–418)	0.271
TLC (10^3^/µL)	7.8 (6–9)	7.9 (6.3–9.9)	0.689
Urea (mg/dL)	25 (21–31)	19.5 (10–27)	0.017
Creatinine (mg/dL)	0.67 (0.4–0.9)	0.4 (0.3–0.5)	0.029
Estimated GFR (ml/min/1.73m^2^)	60 (43–82)	101 (74–116)	< 0.001
AST (U/L)	23 (16–31)	15.5 (12–25)	0.024
ALT (U/L)	24 (12–28)	18 (13–27)	0.865
pH	7.3 (7.26–7.37)		
Fasting blood glucose (mg/dL)	90 (84–96)		
Ammonia (mg/dL)	100 (85–117)		
Lactate (mg/dL)	19 (15–32)		
C3 at recruitment (umol/L)	8.1 (7.7–15.35)		
C3/C2 at recruitment	0.37 (0.31–0.43)		

*Hb* hemoglobin, *PLT* platelets, *TLC* total leucocytic count, *ALT* alanine transaminase, *AST* aspartate transaminase, *C3* propionyl carnitine, Data was summarized as median and inter-quartile ranges (IQR) for numerical data and numbers and percentages for categorical data. Mann-Whitney’s U-test was used to assess the difference of quantitative variables, while Chi-Square test was used for categorical variables. *P*-values < 0.05 were considered statistically significant

The clinical features of MMA children were diverse and multisystemic with 63.1% of patients having neurological manifestation, such as seizures, irritability, disturbed consciousness and lethargy, 63.1% had gastrointestinal manifestations, such as vomiting and recurrent/persistent diarrhea, while metabolic manifestations, such as, metabolic acidosis, hyperammonemia were present in 47.4% of patients, 15.7% had severe failure to thrive and 10% had cardiac complications. Among patients there were four children (21.1%) that were well-controlled on treatment while 78.9% were with suboptimal metabolic control. Children with suboptimal metabolic control commonly had one or more of the following features during clinical examination: delayed motor development and/or intellectual disability, abnormal gait, extrapyramidal manifestation, poor academic performance, optic atrophy, cardiomyopathy and chronic kidney disease (Table 1).

Regarding radiological findings 15.8% of MMA children had mild brain atrophy revealed by brain MRI and cranial US, 10.5% of patients had cardiomyopathy determined by echocardiography and 5.3% had hepatomegaly determined by abdominal US.

Comparing the two groups revealed a significant increase in urea, creatinine, and AST in MMA patients compared to controls (*P* = 0.017, 0.029 and 0.024, respectively). Hemoglobin was also significantly decreased in patients compared to controls (*p* < 0.001) (Table 1).

Propionyl carnitine (C3) levels were initially elevated in blood spots of all patients and methylmalonic, 3-hydroxypropionic and 2-methylcitric acids were also elevated in the urine of all recruited children as confirmed by GCMS.

### Oxidative stress biomarkers

Comparing patients and control groups concerning the screened biomarkers revealed significant increases in FGF21 and GDF15 in MMA patients compared to controls (*P* = 0.037 and < 0.001, respectively) and significant decreases in total glutathione, reduced glutathione and reduced/oxidized glutathione (GSH/GSSG) ratio (*P* < 0.001 for all) (Table 2).

**Table 2:** Comparing oxidative stress biomarkers levels between MMA patients and healthy controls and between complicated and non-complicated MMA patients

Variable	Patients group (*n* = 19)		Control group (*n* = 30)	*P* value
FGF21 (pg/ml)	127 (41–460)		54 (24–117)	0.037
GDF15 (pg/ml)	145 (72–168)		49 (22–94)	< 0.001
Total Glutathione (µmol/L)	2.6 (2.0–4.0)		7.6 (5.5–8.1)	< 0.001
Reduced Glutathione (µmol/L)	0.61 (0.14–1.32)		4.8 (3.47–5.92)	< 0.001
GSH/GSSG	0.28 (0.08 − 0.47)		2.26 (1.46 − 10.31)	< 0.001

	**Complicated** (**n** = 5)	Non-complicated (*n* = 14)		***P*** ** value**
FGF21 (pg/ml)	1952 ± 1515	144 ± 154		0.038
GDF15 (pg/ml)	257 ± 225	114 ± 66		0.149
Total Glutathione (µmol/L)	2.87 ± 1.34	3.36 ± 1.73		0.960
Reduced Glutathione (µmol/L)	0.57 ± 0.81	0.98 ± 1.06		0.289
GSH/GSSG	0.17 ± 0.18	0.34 ± 0.3		0.294

*FGF21* Fibroblast growth factor 21, *GDF15* growth differentiation factor 15, *GSH/GSSG* reduced/oxidized glutathione ratio. Data were summarized as median and inter-quartile ranges (IQR) or as mean and standard deviations. Mann-Whitney’s U-test was used to assess the difference of quantitative variables. *P*-values < 0.05 were considered statistically significant

When comparing the oxidative stress biomarkers levels in children with multisystem involvement, such as with renal, cardiac or hepatic affection (*n* = 5), versus children having only metabolic and neurological manifestations (*n* = 14), FGF21 showed a significant difference 1952 ± 1515 pg/ml vs. 144 ± 154 pg/ml, respectively; *P* = 0.038. GDF15 showed a trend for elevation in complicated children 257 ± 225 pg/ml vs. 114 ± 66 pg/ml in the non-complicated; however, it didn’t reach statistical significance (*P* = 0.149) (Table 2).

We further evaluated the correlation between the tested biomarkers levels in patients’ group and their potential predictors of clinical and laboratory data. In this analysis only serum ammonia had a significant negative correlation with total glutathione in the patient group (*r*= ‒0.468, *P* = 0.027). Other clinical and laboratory parameters didn’t show direct significant correlations with biomarkers levels.

When correlating the various biomarkers of oxidative stress with each other FGF21 and GDF15 had no significant correlation (*P* = 0.301). However, GDF15 had a strong negative correlation with each total glutathione, reduced glutathione and reduced/oxidized glutathione ratio (GSH/GSSG) (*P* < 0.001, *P* < 0.001 and *P* = 0.005, respectively). In contrast FGF21 had only a significant negative correlation with the GSH/GSSG ratio (*P* = 0.038) (Table 3).

**Table 3:** Correlations among different biomarkers of oxidative stress in both MMA patients and control groups (*n*=49)

	Total glutathione	Reduced glutathione	GSH/GSSG	FGF21	GDF15
Total glutathione	-------	*r*= 0.917 *P*<0.001	-------	*r*= –0.063 *P*= 0.334	*r*= –0.443 *P*< 0.001
Reduced glutathione	*r*=0.917 *P*<0.001	-------	-------	*r*= –0.119 *P*= 0.208	*r*= –0.459 *P*< 0.001
GSH/GSSG	-------	-------	-------	*r*= –0.256 *P*= 0.038	*r*= –0.367 *P*= 0.005
FGF21	*r*= -0.063 *P*= 0.334	*r*= –0.119 *P*= 0.208	*r*= –0.256 *P*= 0.038	-------	*r*= –0.076 *P*= 0.301
GDF15	*r*= –0.443 *P*< 0.001	*r*= –0.459 *P*< 0.001	*r*= –0.367 *P*= 0.005	*r*= –0.076 *P*= 0.301	-------

*FGF21* fibroblast growth factor 21, *GDF15* growth differentiation factor 15, *GSH* reduced glutathione, *GSSG* oxidized glutathione. Spearman's correlation coefficient was used for correlation analyses

During the forward-model multivariate linear regression analysis, FGF21 was an independent negative predictor of the estimated GFR values in MMA patients (t=‒7.270; *P* < 0.0001), together with the duration of illness (t=‒4.384; *P* = 0.0006), ammonia (t=‒2.520; *P* = 0.024) and fasting glucose (t=‒2.604; *P* = 0.02). Other oxidative biomarkers were not significant predictors for eGFR in the model. And when evaluating the predictors of blood hemoglobin as the dependant variable in the second linear regression model, only plasma fasting glucose was an independent predictor (t=‒2.690; *P* = 0.015) (Table 4). Multivariate logistic regression analysis was also performed with the presence of complications as a dichotomous dependent variable; however, none of the oxidative biomarkers were independently associated with the risk of complications.

**Table 4:** Multivariate regression models for predicting the independent factors affecting eGFR, hemoglobin and presence of complications in MMA children in our study

Predictor Factor	β	t	*P* value
I- Dependent variable: eGFR. Multivariate linear regression model for the factors affecting eGFR. Independent variables: Age at recruitment, duration of illness, sex, ammonia, lactate, fasting glucose, ALT, hemoglobin, FGF21, GDF15, Total glutathione, reduced/oxidized glutathione ratio			
(Constant)	-75.6082		
Duration of illness	-0.3969	-4.384	0.0006
Ammonia	-0.3227	-2.520	0.024
Fasting glucose	-1.1064	-2.604	0.02
FGF-21	-0.02657	-7.270	< 0.0001
II- Multivariate linear regression for the factors affecting blood hemoglobin Dependent variable: Blood hemoglobin Independent variables tested: Age at recruitment, duration of illness, sex, ammonia, lactate, fasting glucose, ALT, creatinine, FGF21, GDF15, Total glutathione, reduced/oxidized glutathione ratio			
(Constant)	17.0089		
Glucose	-0.07301	-2.690	0.015

Variables with P value > 0.1 were excluded from the models. Β: regression coefficient, eGFR: estimated glomerular filtration rate, t: relative difference

### ROC curve analysis

**Fig. 1:**
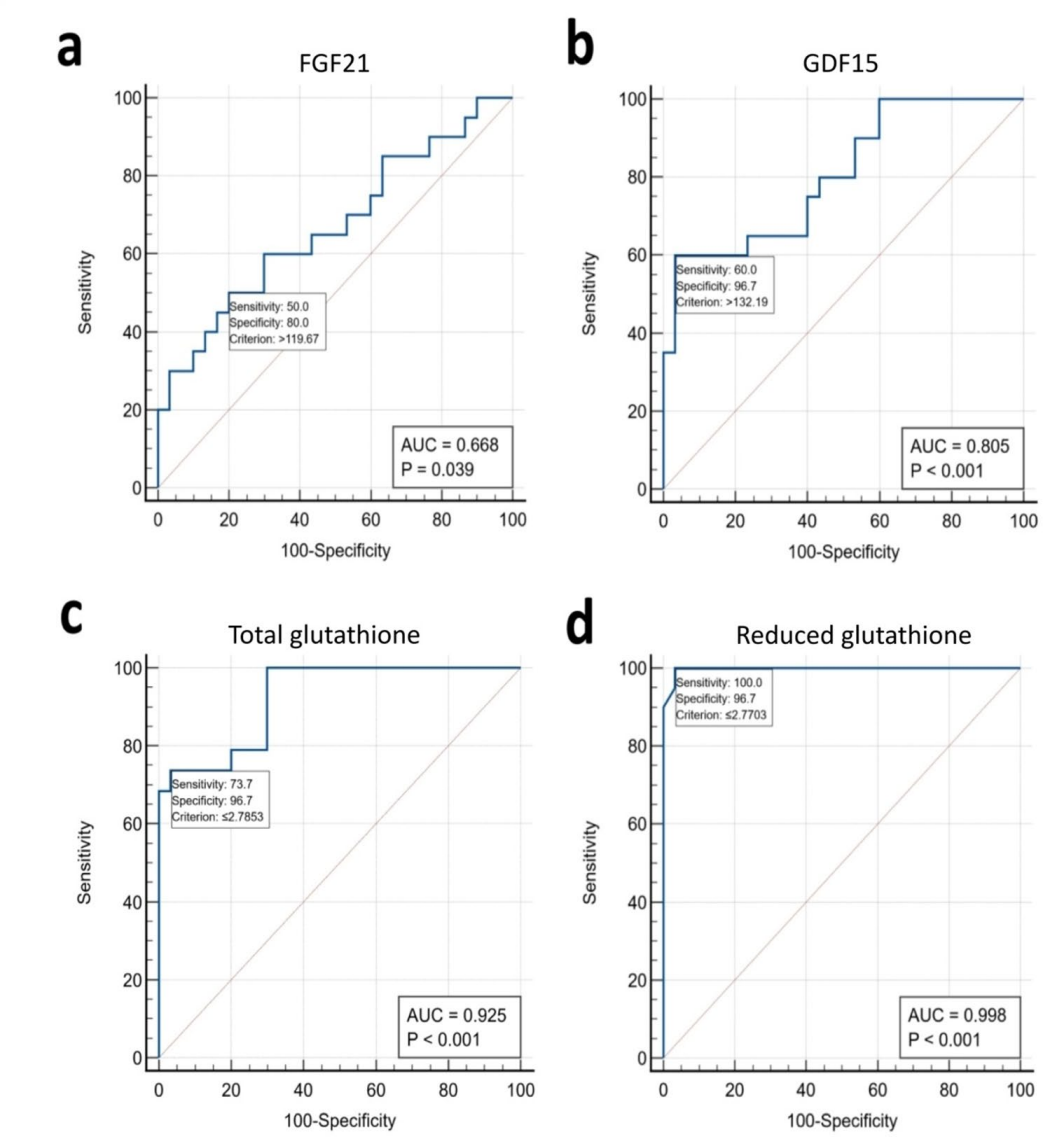
Receiver Operating Characteristic (ROC) curve analysis of the tested biomarkers comparing their levels in MMA patients and healthy controls: (**a**) FGF21, (**b**) GDF15, (**c**) Total glutathione and (**d**) Reduced glutathione

In the ROC curve analysis we evaluated the performances of different oxidative stress biomarkers in MMA patients vs. controls; the area under the curve (AUC), best cut off, sensitivity, specificity and negative and positive predictive values were calculated (Fig. 1).

FGF21 at a cutoff point of 119.7 pg/ml predicted patients with MMA with 50% sensitivity, 80% specificity, 62% and 72% for positive and negative predictive values, respectively and AUC of 0.668 (*P* = 0.039). GDF15 at a cutoff point of 132.2 pg/ml has a much better performance with 60% sensitivity, 96% specificity, 91% and 78% for positive and negative predictive values, respectively and AUC of 0.805 (*P* < 0.001). The glutathione based biomarkers were performing better under the ROC curve analysis, especially the GSH/GSSG ratio (Table 5).

**Table 5:** Performance characteristics of the tested oxidative stress biomarkers

	FGF21 (pg/ml)	GDF15 (pg/ml)	Total glutathione (µmol/L)	Reduced glutathione (µmol/L)	GSH/GSSG
AUC	0.668	0.805	0.928	0.998	1.0
*P*-value	0.039	< 0.001	< 0.001	< 0.001	< 0.001
Optimal cut-off	119.7	132.2	2.78	2.57	0.93
Sensitivity	50%	60%	73	100	100
Specificity	80%	96%	96	96	100
PPV	62%	91%	93	95	100
NPV	72%	78%	85	100	100

*AUC* area under the ROC curve, *FGF21* fibroblast growth factor 21, *GDF15* growth differentiation factor 15, *GSH* reduced glutathione, *GSSG* oxidized glutathione, *ROC curve* receiver operating characteristic curve, *NPV* negative predictive value, *PPV* positive predictive value. The highest Youden-Index was used to choose the optimal cut-off points

## Discussion

Methylmalonic acidemia is among the most prevalent autosomal recessive organic acidemias and is characterized by the build-up of methylmalonic acid due to deficiency of one of the enzymes involved in the metabolic pathways of propionyl-CoA to produce succinyl-CoA for the Krebs cycle [8]. The resulting oxidative stress depletes antioxidants like glutathione, leading to cellular damage through lipid peroxidation, protein modification, and DNA injury. High-energy-demand tissues as the brain and kidneys are especially vulnerable for these defects, thus the child commonly manifests with developmental delay, seizures, chronic renal dysfunction and other multi-organ pathology [19].

In the studied Egyptian cohort, patients’ age ranged between 1.2 and 14 years at recruitment and the age of onset of symptoms ranged between 3 days to 16 months (median 5.6; IQR 1.25–6 months). The age of diagnosis ranged between 1 and 36 months (median 14.4; IQR 2.5–16 months). This signifies the relatively long odysseys that most MMA children suffered before diagnosis (median 6 months, range 1–30 months). Similar recent studies in the Middle East reported approximately the same ages for presentation and diagnosis of MMA children [20, 21]. The establishment of a routine newborn screening program that includes methylmalonic acidemia and other organic acid disorders in Egypt will minimize this diagnostic delay and will give a better therapeutic outcome when affected newborns start their treatment regimens early before the development of irreversible disease complications [4, 5, 22].

Consanguinity remains highly prevalent in the Middle East and North Africa (MENA) region, with rates ranging from 20% to over 50% in some countries [23]. In this study the consanguinity rate was 73.7% among the affected families. Consanguinity rates for MMA patients recruited in countries of the region, such as Iran and Syria are comparably in the range of 70–85% as well denoting the impact of this social practice on the incidence of autosomal recessive disorders, such as MMA [21, 24, 25].

Methylmalonic acidemia is reported as the most frequent OA disorder in many Arab countries such as Tunisia [26], Lebanon [27] and Syria [25]. It is also frequently identified as the most common OA in several Asian countries [28]. Neurological manifestations, such as seizures, developmental delay, movement disorders and encephalopathy and metabolic manifestations, such as acidosis, hyperammonemia were dominant in the majority of our patients, which is similar to cohorts from both developed and developing countries [26, 29–32].

Although there are multiple genes that are potentially responsible for the genetic background of our MMA cohort, oxidative stress is expected to play a significant role in the pathogenesis of all genotypes. Both the levels of the tested biomarkers FGF21 and GDF15 were significantly elevated in MMA patients compared to healthy controls (*P* = 0.037 and < 0.001, respectively). Tsygankova et al., 2019 suggested that FGF21 and GDF15 levels increase in patients with mitochondrial dysfunction and oxidative stress and detected no clear correlation between the severity of the disease status and FGF21 or GDF15 levels in the blood of affected patients [33].

Although MMA is not a primary mitochondrial disorder, the role of mitochondrial dysfunction and oxidative stress in the pathogenesis of MMA cannot be overestimated. This is clear with the significantly reduced levels of total and reduced glutathione in all MMA patients compared to controls. When correlating the blood levels of FGF21 and GDF15 to established biomarkers of oxidative stress, such as reduced glutathione and reduced/oxidized glutathione ratio (GSH/GSSG), both biomarkers showed a very good correlation with GSH/GSSG ratio (*P* = 0.038 and 0.005, respectively). However, only GDF15 showed a significant correlation with both reduced and total glutathione levels (*P* < 0.001 for both) (Table 3). This may indicate that GDF15 outperforms FGF21 as a representative of oxidative stress in MMA children. On the other hand, FGF21 in the linear regression analysis was the only tested oxidative biomarker to have a significant inverse correlation with estimated GFR values, which means it is a better predictor of renal deterioration in MMA patients.

Concerning ROC curve analysis for the separation of MMA patients vs. controls, FGF21 and GDF15 showed area under the curve (AUC of 0.668 and 0.805, respectively), with overall sensitivity of 50% and 60%, and specificity of 80% and 96%, respectively and a better performance from GDF15. This was in agreement with the meta-analysis performed by Lin et al., 2020 who studied mitochondrial disorders. They reported that GDF15 was more sensitive and specific than FGF21 for the detection of mitochondrial impairment [34]. In contrast, more recent data reported by Riley et al., 2022 in 56 pediatric patients with mitochondrial disease compared to 104 with non-mitochondrial disease suggested that FGF21 outperformed GDF15 as a screening biomarker [35].

Although total and reduced glutathione and the GSH/GSSG ratio all have excellent performances under the ROC curve analysis for differentiating MMA patients from controls in our study (AUC: 0.925, 0.998 and 1, respectively), their sample stability is low and their required storage conditions are more demanding compared to the more stable proteins FGF21 and GDF15 which were easily stored and analyzed. GDF15 particularly has a good performance under the ROC curve and thus can be considered as a screening biomarker for the evaluation of oxidative stress in MMA.

The results of our study should be observed within the context of the limitations of our experimental design. One limitation was the overall sample size, which is restricted by the rarity of the syndrome. Correlations with clinical and laboratory parameters may have also been restricted by the limited sample size. We did not perform genetic confirmation for the patients diagnosed at our center due to limited resources; however, the dual biochemical confirmation by tandem mass spectrometry and gas chromatography mass spectrometry should leave no space for doubt regarding MMA diagnosis. Minor differences in the pathogenic pathways of each specific gene defect in any of our MMA children hypothetically could alter the levels of oxidative stress and hence the tested biomarkers; however, we believe this effect would be minimal as the impaired redox mechanisms is expected to be evident in all genotypes. Furthermore, getting a conclusive difference in different genotypes would require the recruitment of a much bigger sample size of MMA patients that can be only achieved through multinational collaboration. Standardized tools for neurological assessment in infants like SINDA (Standardized Infant NeuroDevelopmental Assessment) [36] and HINE (Hammersmith Infant Neurological Examination) [37] or other advanced quantitative neuropsychological testing could be more informative when used as evidence for disease severity when compared with the oxidative stress biomarkers. Finally, the study is cross sectional, thus the association of biomarkers levels with disease progression in confirmed cases cannot be properly evaluated.

In conclusion, methylmalonic acidemia is a serious neurometabolic disorder that lacks feasible and practical tools for evaluation of oxidative stress and clinical severity scoring needed for future therapeutic trials. Both evaluated biomarkers GDF15 and FGF21 were significantly elevated in the blood of MMA children, although GDF15 had a much better performance compared to the poor performance of FGF21 under the ROC curve analysis. In contrast, FGF21 was superior in predicting kidney function deterioration in MMA patients. Furthermore, both biomarkers had a significant negative correlation with GSH/GSSG ratio, and GDF15 had also a significant negative correlation with reduced and total glutathione levels. Upon further longitudinal studies, both biomarkers seem promising candidates for the use as potential oxidative stress biomarkers in MMA.

## Data Availability

All data generated or analyzed during this study are included in the published article. Any further data are available from the corresponding author upon reasonable request.
